# Exercise May Ameliorate the Detrimental Side Effects of High Vitamin D Supplementation on Muscle Function in Mice

**DOI:** 10.1002/jbmr.3985

**Published:** 2020-03-11

**Authors:** Danielle A Debruin, Cara A Timpani, Hannah Lalunio, Emma Rybalka, Craig A Goodman, Alan Hayes

**Affiliations:** ^1^ Australian Institute for Musculoskeletal Science (AIMSS), Victoria University, Sunshine Hospital St Albans Australia; ^2^ Institute for Health and Sport, Victoria University Melbourne Australia; ^3^ Department of Medicine ‐ Western Health Melbourne Medical School, The University of Melbourne Melbourne Australia; ^4^ Centre for Muscle Research (CMR), Department of Physiology The University of Melbourne Parkville Australia

**Keywords:** CHOLECALCIFEROL, MITOCHONDRIA, MUSCLE FATIGUE, MUSCLE FUNCTION, VITAMIN D, VOLUNTARY RUNNING

## Abstract

Vitamin D is commonly prescribed to normalize deficiencies and to treat osteoporosis. However, the effect vitamin D supplements have on skeletal muscle health is equivocal. Although vitamin D is known to play a role in the various processes that maintain muscle integrity and function, recent studies utilizing high bolus dose vitamin D supplementation has demonstrated an increased risk of falls. Thus, the aim of this study was to investigate the effects of high vitamin D supplementation on skeletal muscle function with and without exercise enrichment. Four‐week old C57BL/10 mice (*n* = 48) were separated into either normal vitamin D (1500 IU/kg diet; unsupplemented) or high vitamin D (20,000 IU/kg diet; supplemented) treatment groups. Each dietary group was further separated into interventional subgroups where mice either remained sedentary or received exercise‐enrichment for 8 weeks in the form of voluntary running. Following the intervention period, whole body in vivo and ex vivo contractile analysis were performed. High vitamin D supplementation decreased force production in the slow‐twitch soleus muscles of sedentary mice (*p* < .01); however, exercise normalized this effect. Eight weeks of exercise did not improve fatigue resistance of the extensor digitorum longus (EDL) or soleus muscles in unsupplemented mice, likely due to low levels of activation in these muscles. In contrast, fatigability was improved in the EDL (*p* < .01) and even more so in the soleus (*p* < .001) in the supplemented exercise‐enriched group. Our data highlights that increasing vitamin D levels above normal reduces postural muscle force as seen in the soleus. Thus, unnecessary vitamin D supplementation may contribute to the increased risk of falls observed in some studies. Interestingly, when vitamin D supplementation was combined with exercise, force production was effectively restored, and fatigue resistance improved, even in muscles lowly activated. Regular exercise may modulate the effects of vitamin D on skeletal muscle, and be recommended for individuals receiving vitamin D supplements. © 2020 The Authors. *Journal of Bone and Mineral Research* published by American Society for Bone and Mineral Research.

## Introduction

Vitamin D is a key hormone that regulates many bodily functions and, importantly, plays a major role in the maintenance of bone and muscle integrity.^1^, ^2^, ^3^, ^4^, ^5^, ^6^, ^7^ For example, vitamin D regulates bone metabolism through the control of mineral ion homeostasis, particularly calcium and phosphate. The association between inadequate levels of vitamin D and skeletal pathologies, such as rickets, are well established.^8^ With respect to skeletal muscle, vitamin D deficiency (commonly reported as serum levels below 50 nmol/L) can lead to, or enhance, skeletal muscle wasting and fatigue—especially in the elderly.^9^, ^10^, ^11^ Importantly, these effects can be attenuated through vitamin D supplementation interventions.^12^ Indeed, studies have shown that vitamin D supplementation in the elderly population with vitamin D deficiency results in improvements in muscle function, such as increased strength and decreased postural sway.^4^, ^5^, ^13^, ^14^ Although many studies have reported that vitamin D supplementation strategies are beneficial in human and rodent models of vitamin D deficiency, there are few studies that have investigated the effects of increasing vitamin D above normal basal levels via supplementation on skeletal muscle function and fatigue.

Although vitamin D supplementation produces the greatest health benefits to deficient individuals when serum levels are normalized, optimal dosages and regimens are still yet to be determined.^4^, ^5^, ^6^, ^10^, ^13^, ^14^, ^15^ Many vitamin D supplementation studies have demonstrated “nonskeletal” benefits, including decreased severity of autoimmune diseases, such as rheumatoid arthritis,^16^, ^17^ and decreased symptoms of depression.^18^ However, research conducted over the past decade has focused on the effects of intermittent high‐dose vitamin D administration consisting of single boluses between 300,000 and 600,000 international units (IU) of vitamin D that are aimed at delivering a yearly dosage (for review see Sanders and colleagues^19^). Even though these studies demonstrated rapid increases in serum 25(OH)D (inactive vitamin D) levels, these annual high doses have been found to be problematic, with reports of increased risk of falls and fracture.^20^, ^21^ Although the mechanisms behind this effect are still unknown, skeletal muscle weakness, leading to increased tripping, has been suggested, and our recent study demonstrated a decrease in ex vivo muscle force production in mice receiving a single yearly dose.^22^

Interestingly, despite potential deleterious effects of high supplementation, vitamin D status has been positively linked with various aspects of muscle function related to greater exercise performance. For example, it has been documented in numerous studies that serum 25(OH)D levels are positively correlated with muscle force and power, jump height, and aerobic capacity.^1^, ^15^, ^23^, ^24^ Although most vitamin D supplementation studies have been conducted on vitamin D–deficient or vitamin D–insufficient humans and animal models, little is known about the potential effects of supraphysiological increases in vitamin D on muscle function and exercise performance. Evidence of improved calcium handling, findings of reduced muscle damage^25^, ^26^ and improved recovery^27^ suggests that increasing vitamin D above sufficient levels may be of added benefit. However, as mentioned above, yearly injections of vitamin D, even in those that are vitamin D–deficient, have been associated with increased risk of falls^20^ potentially linked with muscle weakness, and similar yearly injections from a replete starting point, albeit in mice, reduced muscle force.^22^ However, this decline was not seen when the same amount was supplemented gradually through the diet. Thus, in this study we investigated whether a gradual administration of high vitamin D would improve exercise performance, and whether there is a synergistic effect of vitamin D and exercise on muscle function.

## Materials and Methods

### Animals and ethics approval

Four‐week‐old C57BL/6J male mice (*n* = 48) were obtained from the Animal Resource Centre (ARC, Western Australia) and housed on a 12‐hour light:12‐hour dark cycle. The mice were fed a standard AIN‐93G diet (Specialty Feeds, Glen Forrest, WA, Australia) from weaning to 4 weeks of age (containing 0.47% calcium, 0.35% phosphate, and vitamin D_3_ [cholecalciferol] 1500 IU/kg). Upon arrival, mice were randomly allocated to dietary groups where they received the same standard AIN‐93G rodent chow (containing 1500 IU/kg vitamin D; UNSUPP; *n* = 24) or a modified AIN‐93G diet containing high vitamin D (20,000 IU/kg feed; Specialty Feeds; VITD; *n* = 24) for 8 weeks. This level of dietary vitamin has previously been shown to elevate serum vitamin D fivefold after 10 weeks of supplementation.^28^ Animals had *ad libitum* access to food and water throughout the treatment period. Animals and food were weighed weekly to enable the estimated calculation of total food and vitamin D consumption per animal/day. Ethics approval was granted for this project by the Victoria University Animal Ethics Committee (project code 13/007) with all experiments abiding by the Australian Code of Practice for the Care and Use of Animals for Scientific Purposes (National Health and Medical Research Council, Australia, 8th edition). To investigate the effects of vitamin D supplementation in combination with exercise, animals from each dietary intervention were randomly divided into two subgroups. For the groups receiving exercise enrichment (EXER UNSUPP [*n* = 12] and EXER VITD [*n* = 12]), mice were housed in Scurry Mouse Activity Wheel Chamber System cages (Model 80820S; Lafayette Instrument Company, Lafayette, IN, USA) with access to a running wheel and constant monitoring of voluntary running behavior throughout the 8‐week protocol. For the sedentary groups (SED UNSUPP [*n* = 12] and SED VITD [*n* = 12]), mice were housed together in groups in standard ventilated cages.

At the conclusion of the 8‐week treatment interventions, mice were scanned for body composition and transferred to metabolic screening cages (Sable Systems International, North Las Vegas, NV, USA) for 48 hours for in vivo analysis of whole‐body metabolism. Subsequently, mice were deeply anesthetized (60 mg/kg sodium pentobarbitone) and muscles of interest were surgically excised. Initially, the fast‐twitch extensor digitorum longus (EDL) and slow‐twitch soleus (SOL) muscles were tied tendon to tendon with 4.0 suture thread followed by immediate excision for analysis of muscle contractile function. Other tissues, including the kidneys and liver, which are the main sites of vitamin D metabolism, were also collected and immediately weighed and snap‐frozen in liquid nitrogen for later analysis.

### Body composition analysis

Prior to the commencement of the 8‐week feeding protocol and immediately after completion, body composition was analyzed using an EchoMRI‐100H scanner (EchoMRI, Houston, TX, USA). Fat, lean, and water mass measurements were taken in triplicate scans with 30‐s time lapses between each. Data is presented as the mean of the three scans and only postinterventional data is shown because no differences were observed in preintervention body composition.

### In vivo analysis of physical activity, behavior, and metabolism

Mice were individually housed in Promethion Metabolic Screening Cages (Sable Systems International, Nth Las Vegas, NV, USA) for 48 hours after the completion of the postintervention EchoMRI body composition scan. Presented data was obtained from the final 24 hours recorded to allow for cage acclimatization. Data points recorded were filtered into 4‐min time intervals using ExpeData PRO Software with a customized macro provided by Sable Systems. All mice were permitted *ad libitum* free access to their respective diets and water, and a voluntary running wheel with continuous recording of interactions. Ambulatory activity and positioning were recorded (*x*, *y*, and *z* axes) within the cages, with non‐wheel activity presented as pedestrian meters. Wheel velocities were used to indicate different working intensities during voluntary exercise.

### Ex vivo detection of muscle contractile function

Ex vivo evaluation of muscle contractile properties was performed as described by us.^29^, ^30^, ^31^ Briefly, excised EDL and SOL muscles were placed into individual organ baths (Danish Myo Technology, Hinnerup, Denmark) filled with Krebs‐Henseleit Ringer's solution (118mM NaCl, 4.75mM KCl, 1.18mM MgSO_4_7H_2_O, 1mM Na_2_HPO_4_, 2.5mM CaCl_2_H_2_O, 24mM NaHCO_3_, and 11mM glucose). Each organ bath was bubbled with carbogen (5% CO_2_/95% O_2_) and maintained at a temperature of 30°C and a pH of 7.4. Data was collected and analyzed using LabChart Pro version 8.0 software, customized for this experiment (ADInstruments, Dunedin, New Zealand). The proximal end of the muscle was attached (via knotted surgical silk loops) onto a previously calibrated force transducer and the distal end was fixed to a micromanipulator with stimulating electrodes flanking the muscle belly. Before the commencement of the contractility experiments, optimal length (L_o_) for each muscle was obtained via a series of twitch contractions at increasing lengths, thereby ensuring optimal overlap of the sarcomeres of each muscle. Muscle length was measured with calipers. A force‐frequency relationship was established by delivering supramaximal square wave pulses (100 Hz, 350 ms and 500 ms duration for EDL and SOL, respectively) at frequencies of 10, 20, 30, 40, 50, 80, 100, 120, 150, and 180 Hz. Absolute force (maximum isometric contraction, P_o_) was recorded as the highest force obtained in the force‐frequency protocol for both muscles, with other forces reported as a percentage of that maximum.

#### Contractile properties

After the force‐frequency relationship was determined, each muscle was stimulated three times over 5 s with a single pulse for the analysis of basic contractile properties (peak twitch force [P_t_], time to peak [TTP], and relaxation time [½RT]). To normalize force production for different muscle sizes, maximal specific force production (sP_o_) was calculated as force produced per cross‐sectional area (CSA). This was calculated based upon L_o_ and muscle mass,^32^ assuming the muscle density is 1.06 g/cm^2^ and fiber‐muscle length ratios are 0.44 for the EDL and 0.71 for the SOL. To investigate muscle fatigability, muscles were exposed to repeated intermittent electrical stimuli for 3 min. The EDL was stimulated every 4 s at 100 Hz for 350 ms and the SOL every 2 s at 80 Hz for 500 ms to obtain comparable levels of fatigue.

### Histological analysis

Following the completion of the contractile protocol, the EDL and SOL muscles were coated in optimal cutting temperature (OCT) compound and snap frozen in liquid nitrogen‐chilled isopentane (Sigma Aldrich, Castle Hill NSW, Australia). The imbedded muscles were cryosectioned (12 μm) and stained using a standard hematoxylin and eosin (H&E) staining protocol (30 s incubation in hematoxylin and 1 m 45 s incubation in eosin).^33^ Slides were mounted with distyrene‐plasticiser‐xylene (DPx) (BDH, Poole, UK), analyzed, and imaged at magnification ×20 using a Zeiss Axio Imager Z2 microscope (Carl Zeiss MicroImaging GmbH, Oberkochen, Germany) as described.^34^ To assess the effects of vitamin D on mitochondrial content in the muscle, a succinate dehydrogenase (SDH) staining procedure was performed on cryosectioned muscle samples (prepared as per H&E staining), as described.^33^, ^34^ After the mounting of slides in glycerol jelly, images were taken at magnification ×20 using the same Zeiss microscope and images were converted to 8‐bit. Color intensity was measured in full cross‐sections of the EDL and SOL with the reference point percentage values set by the first control image analyzed with ImageJ software (NIH, Bethesda, MD, USA; https://imagej.nih.gov/ij/).

Staining for muscle fat was conducted as described.^34^ Briefly, SOL 12‐μm sections were air dried and fixed in 3.7% formaldehyde for 60 min followed by rinsing with deionized water baths in triplicate. Following incubation in Oil Red O (ORO) working solution (5:1 ORO in 60% triethyl phosphate [Sigma‐Aldrich, Castle Hill, NSW, Australia]) for 30 min. Slides were washed and mounted with 10% glycerol in PBS. Full cross‐section images were converted to 8‐bit photos with a consistent threshold percentage applied, then analyzed by assessing the intensity of black to white.

Muscle calcification was determined via Alizarin red staining as described.^33^ Frozen SOL sections were cut as per ORO staining and was assessed using Alizarin Red (TMS‐008‐C; Merck Millipore), a dye that chelates with calcium to form Alizarin Red S‐Ca2+ complexes. Slides were stained in the dye for 2 min and dipped in 100% acetone 20 times. The final step included dipping the sections in acetone‐xylene (1:1) 20 times, before being placed in xylene for 1 min. Samples were mounted with DPx, then the whole cross‐section was imaged and analyzed following the same steps as the ORO sections. Data are expressed as arbitrary units.

### Citrate synthase activity

All samples were analyzed for citrate synthase (CS) activity as a marker of mitochondrial density as described.^33^ Thawed tibialis anterior (TA) muscles were homogenized (TissueLyser; Qiagen, Hilden, Germany) for 20 s in 2 mL ice‐cold Western blot (WB) buffer (40mM Tris, pH 7.5; 1mM ethylenediaminetetraacetic acid; 5mM ethylene glycol tetraacetic acid [EGTA]; 0.5% Triton X‐100; 25mM β‐glycerophosphate; 25mM NaF; 1mM Na3VO4; 10 μg/mL leupeptin (LEU); and 1mM phenylmethylsulfonyl fluoride [PMSF]). An aliquot of homogenized sample was added to reagent cocktail (containing 100mM Tris Buffer, 1mM 5,5'‐dithiobis‐(2‐nitrobenzoic) acid [DTNB], and 3mM acetyl coenzyme A) and CS activity was measured spectrophotometrically (Bio‐Rad Laboratories, Hercules, CA, USA; 412 nm at 25°C) for 5 min following the addition of 10mM oxaloacetate. CS activity was calculated using the extinction coefficient of 13.6.^35^ CS activity was normalized to whole‐muscle protein concentration.

### Western blot analysis

Briefly, frozen tissues were homogenized for 20 s in 2 mL ice‐cold Western Immunoprecipitation Kinase (WIK) buffer (40mM Tris‐HCl, pH 8.5; 1mM EDTA, pH 8.5; 5mM EGTA, pH 8.8; 0.5% Triton X‐100; 600mM B‐glycerophosphate; 1M NaF; 0.2M PMSF; 10 mg/mL LEU; and 1M Na_3_VO_4_). Samples were separated into 1‐mL aliquots and stored at −80°C until further analysis. Sample protein concentrations were determined with a detergent compatible (DC) protein assay kit (Bio‐Rad Laboratories, Hercules, CA, USA), and equivalent amounts of protein (30 μg) from each sample were dissolved in 2× SDS sample buffer (20% glycerol; 100mM Tris, pH 6.8; 4% SDS; 0.017% bromophenol blue; 0.25M dithiothreitol [DTT]) and boiled for 5 min at 95°C. Samples were subjected to electrophoretic separation on 7.5% to 12% SDS‐PAGE acrylamide gels as described.^36^ Proteins were then transferred to a polyvinylidene difluoride (PVDF) membrane in transfer buffer (242mM Tris, 58mM glycine, and 20% methanol). The membranes were blocked with 5% skim milk in TBST (Tris‐buffered saline with 0.1% Tween20) for 1 hour and incubated overnight at 4°C with the primary antibody of interest (see Table 1 for antibody details) dissolved in TBST containing 1% BSA. The Total Oxidative Phosphorylation (OXPHOS) Antibody Cocktail consisted of primary antibodies against the following proteins: NADH dehydrogenase (ubiquinone) 1 beta subcomplex, 8 (CI), succinate dehydrogenase assembly factor 4 (CII), ubiquinol‐cytochrome‐C reductase complex core protein 2 (complex III [CIII]), mitochondrially encoded cytochrome C oxidase I (CIV), and mitochondrial ATP synthase subunit alpha (complex V [CV]). Membranes were then washed in TBST for 30 min and incubated for 1 hour at room temperature in 5% skim milk/TBST containing anti‐rabbit horseradish peroxidase conjugated IgG (heavy and light chains [H + L]) antibody (1:5000). Blots were developed with a DARQ charge‐coupled device (CCD) camera mounted to a Fusion FX imaging system (Vilber Lourmat, Eberhardzell, Germany) using ECL Prime reagent (Amersham Biosciences, Piscataway, NJ, USA). After image capture membranes were stained with Coomassie Blue to ensure equal loading in all lanes. To account for minor variations in loading, the signal from the protein of interest was normalized to the Coomassie Blue intensity.

**Table 1:** Antibody List

Antibody	Supplier	Catalogue #	Dilution
CYP24A1 antibody rabbit (polyclonal)	ProteinTech	21582‐1‐AP	1:1000
CYP27B1 antibody rabbit (monoclonal)	Abcam	AB206655	1:1000
Vitamin D Binding protein ‐ antibody rabbit (polyclonal)	Sigma	HPA019855	1:500
Cytochrome c (CytC) (D18C7) antibody rabbit (mAb)	Cell Signaling	11940	1:1000
Oxidative Phosphorylation (OXPHOS) Antibody Cocktail	Abcam	AB110413	1:1000

### Statistical analysis

Data are presented as mean ± standard error of the mean (SE) unless stated otherwise. A two‐way analysis of variance (ANOVA) was used with supplementation (UNSUPP versus VITD) and exercise enrichment (EXER versus SED) as fixed factors, and a post hoc Tukey's test was conducted when there was an interaction. Statistical analysis was conducted with the use of Prism 7.0 software (GraphPad Software, La Jolla, CA, USA). Alpha was set at 0.05 and *p* values between .05 and .10 were considered a trend. Animals were randomly allocated to a treatment group upon acclimatization and histological analysis performed in a blinded fashion.

## Results

### Body weight, food consumption, and body composition

**Figure 1:**
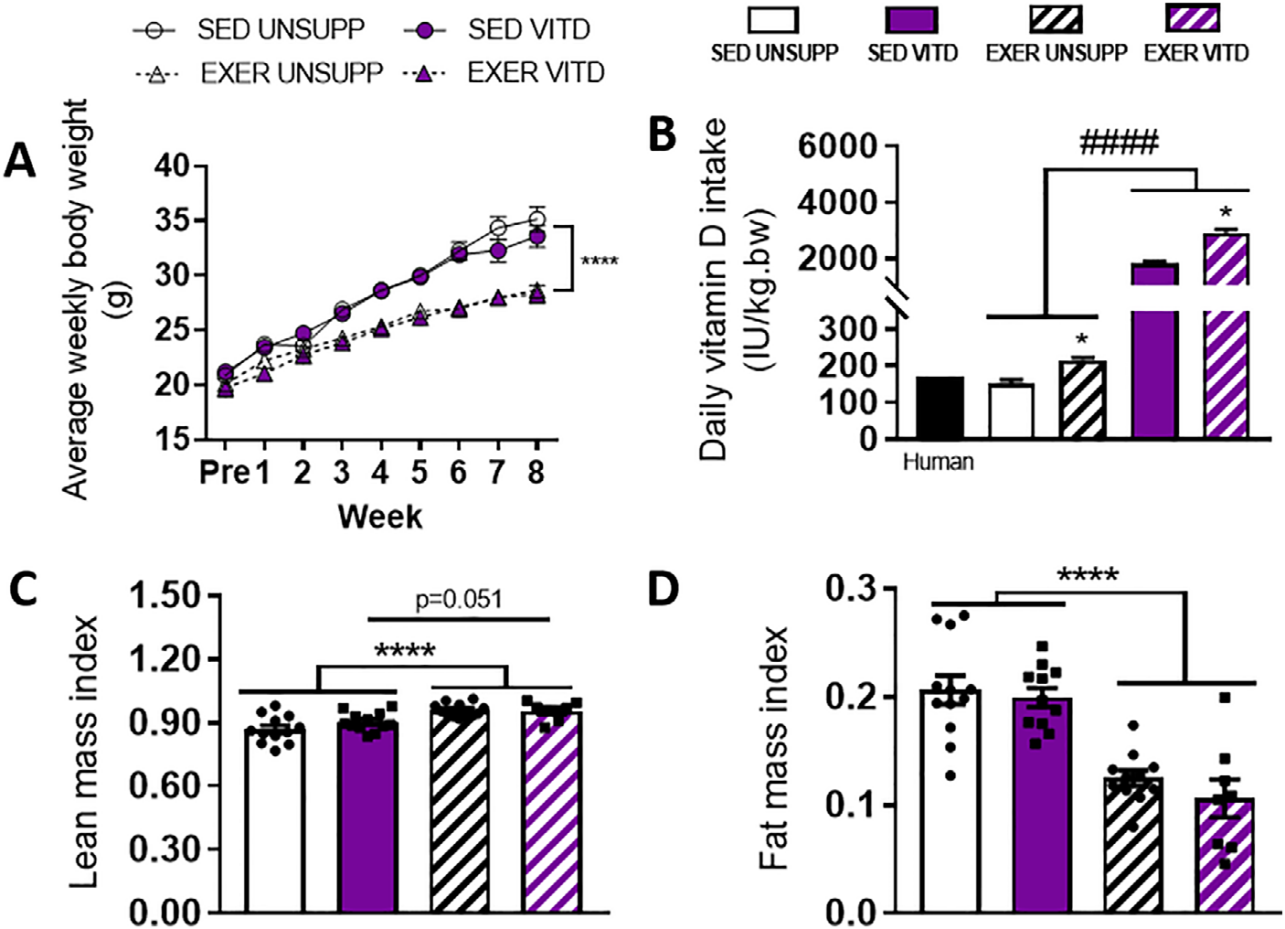
Weekly body weight, daily vitamin D intake, and postintervention body composition of UNSUPP and VITD SED and EXER mice. Weekly body weight for all groups (*A*), daily vitamin D intake expressed as IU/kg (*B*) recommended daily VITD intake of 1000 IU for an average 70‐kg human (solid black bar) converted to the equivalent for a 20‐g mouse based upon Kn factor^37^ and body weight. Body composition analysis for lean mass (*C*) and fat mass (*D*). ####*p* < .0001, vitamin D effect; *p < .05 and ****p < .0001, exercise enrichment effect. *n* = 10–12 per group. EXER = exercise‐enriched; SED = sedentary; UNSUPP = vitamin D–unsupplemented; VITD = vitamin D–supplemented.

Body weight and food consumption were monitored weekly. As shown in Fig. 1, there was no effect of vitamin D on body weight at any time point during the 8‐week treatment regimen. Not surprisingly, the exercise enriched mice weighed significantly less than the sedentary controls (*p* < .0001, Fig. 1 *A*), despite consuming more food on an average weekly basis (*p* < .05, Fig. 1 *B*). To assess the effect of vitamin D with and without exercise enrichment on total body composition, postintervention EchoMRI scans for lean (Fig. 1 *C*) and fat mass (Fig. 1 *D*) were conducted. A separate *t* test revealed a trend for vitamin D to increase lean mass composition in sedentary mice (SED UNSUPP versus SED VITD; *p* = .051, Fig. 1 *C*). As expected, the exercise‐enriched groups had a significantly greater lean mass than the sedentary groups, demonstrating a clear effect of exercise enrichment on increasing lean mass (*p* < .0001, Fig. 1 *C*). There was a clear main effect of exercise enrichment on reducing fat mass (*p* < .0001, Fig. 1 *D*) with no influence of vitamin D.

### Correlations between VITD intake and body composition indices

**Figure 2:**
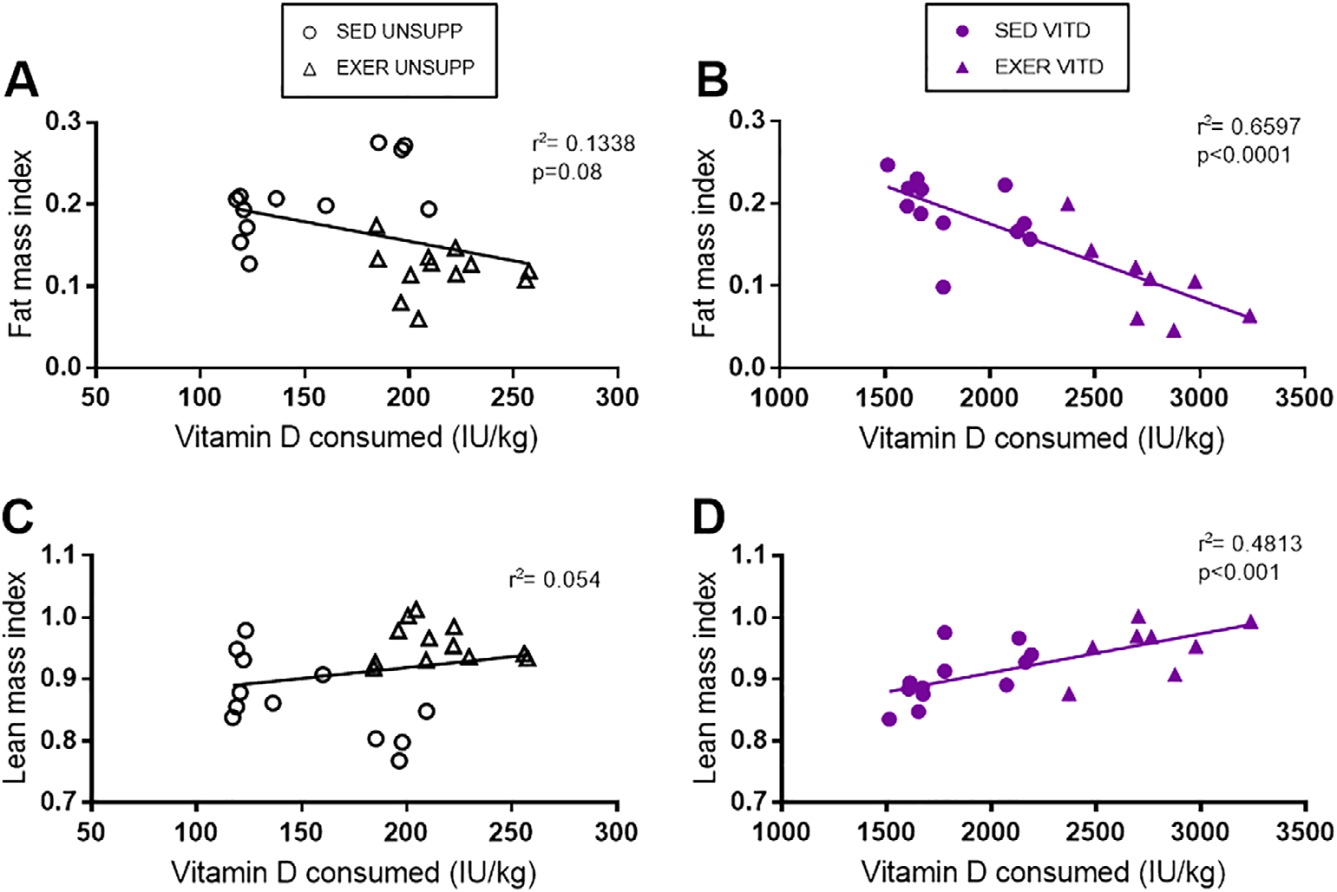
Correlations between total vitamin D consumed and body composition of UNSUPP and VITD SED and EXER mice. Lean mass correlations with total vitamin D consumed for UNSUPP mice (*A*) and VITD mice (*B*) SED and EXER animals. Fat mass correlations with total vitamin D intake for UNSUPP (*C*) and VITD (*D*) SED and EXER mice. Symbols: black open circle, SED UNSUPP; black open triangle, EXER UNSUPP; blue full circle, SED VITD; and blue full triangle, EXER VITD. *n* = 12 per group. EXER = exercise‐enriched; SED = sedentary; UNSUPP = vitamin D–unsupplemented; VITD = vitamin D–supplemented.

To take into account the variability of vitamin D intake between mice and groups, and the effect it may elicit on body composition, correlations were determined between the total amount of vitamin D consumed over the 8 weeks and body composition (lean and fat mass) for each animal. It was found that there was no significant relationship between lean or fat mass with vitamin D intake or exercise enrichment in the UNSUPP animals (Fig. 2 *A*,*C*). However, it was interesting to note that strong correlations were found in the VITD animals only regardless of exercise status. It appears that increased vitamin D intake increases lean mass index (*p* < .001, Fig. 2 *B*) and decreases fat mass index (*p* < .0001, Fig. 2 *D*).

### Organ and muscle weight

There were no differences in body weight upon arrival between all groups. Whole body weight changes were observed with the EXER UNSUPP and EXER VITD mice having a significantly smaller total body weight posttreatment compared to SED UNSUPP and SED VITD mice, which was accompanied with a lower overall weight gain (*p* < .0001, Table 2). After all in vivo analyses were completed, the EDL, SOL, and TA hind limb muscles were harvested and weighed to assess the effect of vitamin D and exercise enrichment on individual muscle masses. Not surprisingly, EDL muscle mass for the EXER UNSUPP and EXER VITD mice was significantly lower when compared to the SED UNSUPP and SED VITD animals (*p* < .001, Table 2) due to a lower overall body weight, such that there were no differences in the muscle mass to body mass ratio (see Fig. 5 *A*,*B*). Interestingly, no changes in muscle weight was observed in the SOL between groups, consistent with an exercise training effect. Indeed, the soleus mass to body mass ratio was significantly higher in the exercised groups (*p* < .0001, Fig. 5 *B*). Vitamin D decreased TA weight in both SED VITD and EXER VITD animals compared to their UNSUPP counterparts (*p* < .05, Table 2). There was a clear exercise enrichment effect on heart and kidney weight, with a significant decrease observed between SED and EXER groups (*p* < .01 and *p* < .05, respectively, Table 2).

**Table 2:** Body, Muscle, and Organ Weights

Parameter	SED UNSUPP	SED VITD	EXER UNSUPP	EXER VITD
BW initial (g)	20.64 ± 1.14	20.57 ± 1.18	20.10 ± 1.63	20.06 ± 1.90
BW final (g)	35.05 ± 2.59	34.06 ± 2.45	28.50 ± 1.64****	27.88 ± 2.09****
BW gain (g)	14.41 ± 2.35	13.98 ± 2.12	8.40 ± 1.69****	7.77 ± 1.83****
EDL (mg)	12.8 ± 0.14	12.9 ± 1.5	11.8 ± 1.7***	11.0 ± 1.3***
SOL (mg)	9.1 ± 0.8	8.6 ± 1.1	9.8 ± 1.7	9.0 ± 1.1
TA (mg)	53.8 ± 10.4	46.6 ± 8.5#	53.0 ± 7.7	49.4 ± 7.4#
Heart (mg)	164.9 ± 13.7	153.8 ± 12.5	140.8 ± 22.7**	135.0 ± 18.8**
Kidneys (mg)	505.7 ± 62.0	610.8 ± 57.5	295.6 ± 25.4*	334.2 ± 54.8*

Results displayed as ± SEM and measured in grams or milligrams. *n* = 8–12 per group. BW = body weight; EDL = extensor digitorum longus; SOL = soleus; TA = tibialis anterior.

**** *p* < .00001

*** *p* < .001

** *p* < .01

* *p* < .05, exercise enrichment effect.

# *p* < .05

vitamin D effect.

### Voluntary exercise capacity and cage activity

**Figure 3:**
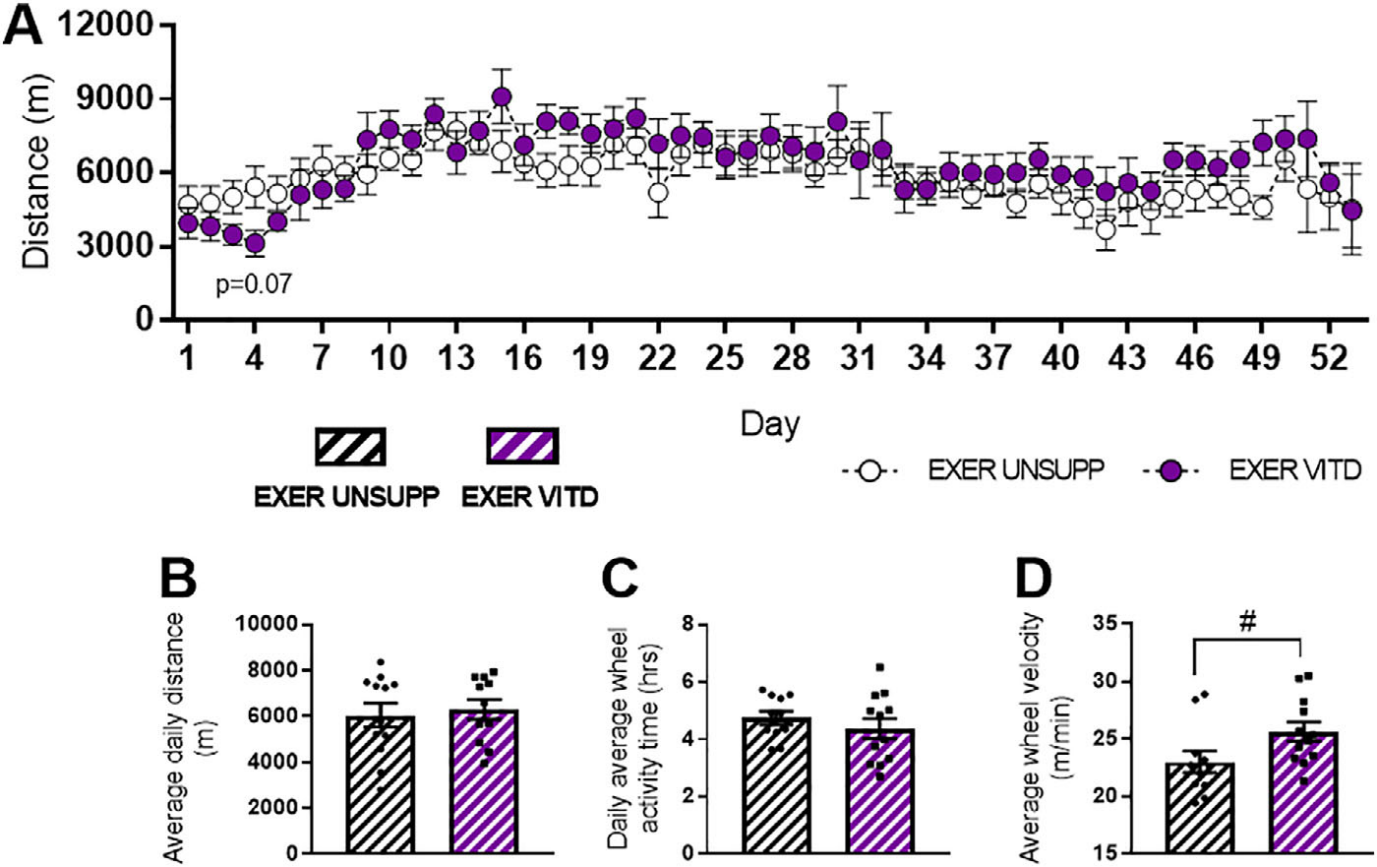
Voluntary running wheel activity during 8‐weeks intervention of UNSUPP and VITD EXER mice. Daily voluntary running distance between UNSUPP and VITD mice (*A*). Average voluntary running parameters measured across the 8 weeks include average daily distance (*B*), time spent on the wheel (*C*), and average wheel velocity (*D*; calculated as average distance/average time). #*p* < .05, vitamin D effect. *n* = 12 per group. EXER = exercise‐enriched; UNSUPP = vitamin D–unsupplemented; VITD = vitamin D–supplemented.

To our knowledge, there are no studies that have combined high vitamin D with voluntary wheel running in rodents to elucidate effects on the skeletal muscular system with exercise. Thus, in addition to assessing the effects of high vitamin D alone, we introduced exercise‐enrichment (access to a voluntary running wheel) during the 8‐week feeding period. We specifically monitored different modalities of voluntary running behavior throughout the intervention including average daily distance, total activity time, and wheel velocity (Fig. 3). Vitamin D did not affect daily voluntary running distance (Fig. 3 *A*), total distance (Fig. 3 *B*), or time spent on the wheel (Fig. 3 *C*); however, it did result in increased wheel velocity (*p* < .05, Fig. 3 *D*). Overall, there was no adverse effect of vitamin D on activity parameters measured. Interestingly, there was an apparent initial decrease in activity over the first 4 days, with a strong trend for less distance covered in the EXER VITD mice on day 4 (*p* = .07, Fig. 3 *A*); however, the mice quickly recovered.

**Figure 4:**
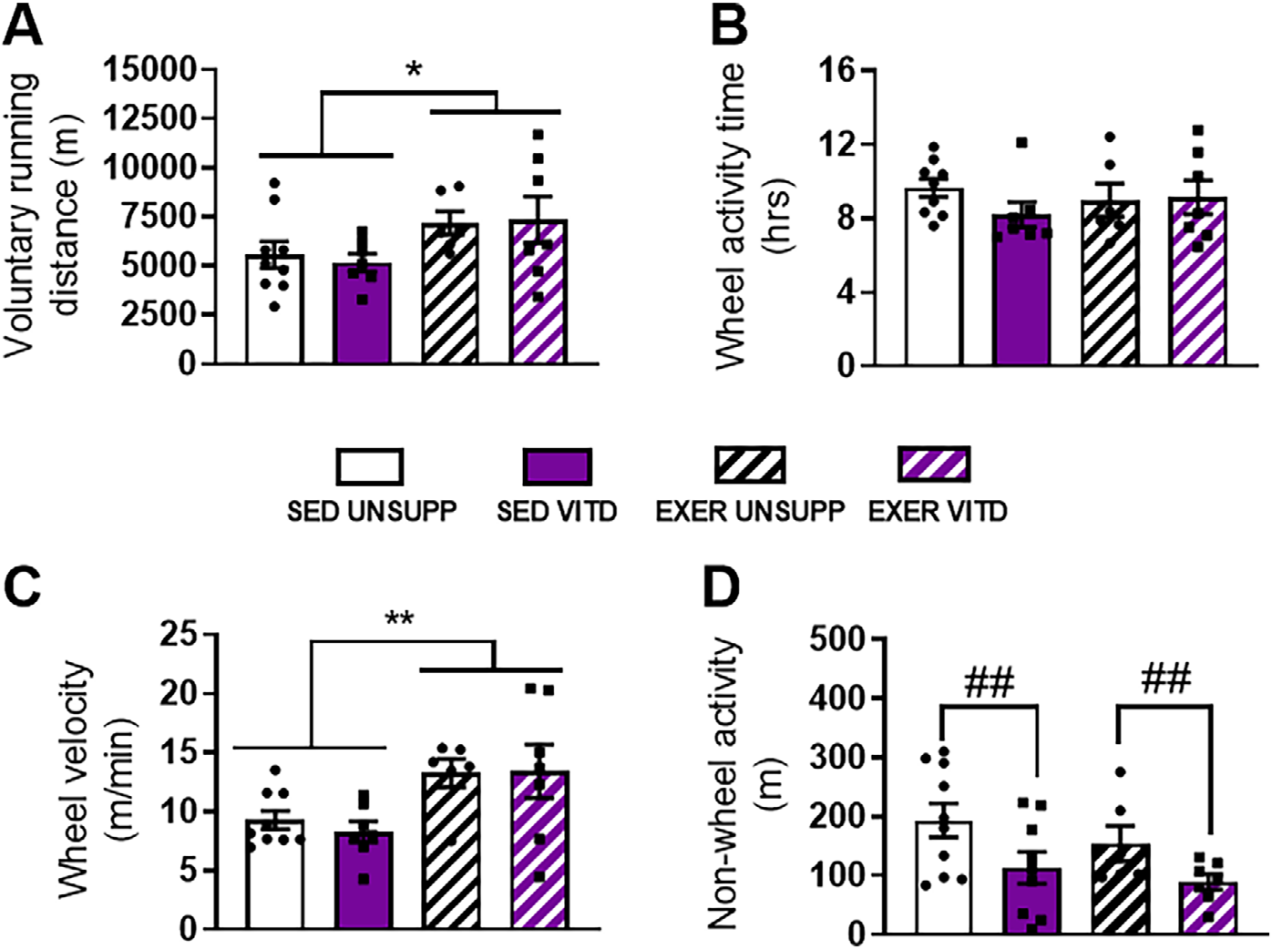
In vivo physical activity measures during 24 hours of Promethion metabolic cage screening of UNSUPP and VITD SED and EXER mice. Total voluntary running distance over the 24‐hour metabolic screening period (*A*), total time spent on the wheel (*B*), and average wheel velocity (*C*) parameters were measured for all groups. (*D*) The amount of distance covered around the cage (non‐wheel/pedestrian meters) was also determined and displayed. *p < .05 and **p < .01; exercise enrichment main effect, ##*p* < .01; vitamin D effect. *n* = 12 per group. EXER = exercise‐enriched; SED = sedentary; UNSUPP = vitamin D–unsupplemented; VITD = vitamin D–supplemented.

To compare the effect of vitamin D on exercise capacity in sedentary and “trained” mice, all animals were screened upon the cessation of the vitamin D feeding and exercise enrichment period via the Promethion Metabolic System for 48 hours. Data was obtained from the last 24 hours of the recording period to allow for acclimatization. There was an overall main effect of exercise enrichment (EXER UNSUPP and EXER VITD compared to SED UNSUPP and SED VITD) to increase wheel distance (*p* < .05, Fig. 4 *A*) despite all groups spending the same amount of time on the wheel (*p* > .05, Fig. 4 *B*). This suggests that the EXER UNSUPP and EXER VITD groups had greater running efficiency, and this was borne out with both groups obtaining wheel speeds that were significantly faster than their untrained sedentary counterparts (*p* < .01, Fig. 4 *C*). Interestingly, vitamin D appeared to decrease non‐wheel activity (pedestrian meters) with decreased meters covered in both SED VITD and EXER VITD mice compared to their UNSUPP controls (*p* < .01, Fig. 4 *D*).

### Contractile parameters

**Figure 5:**
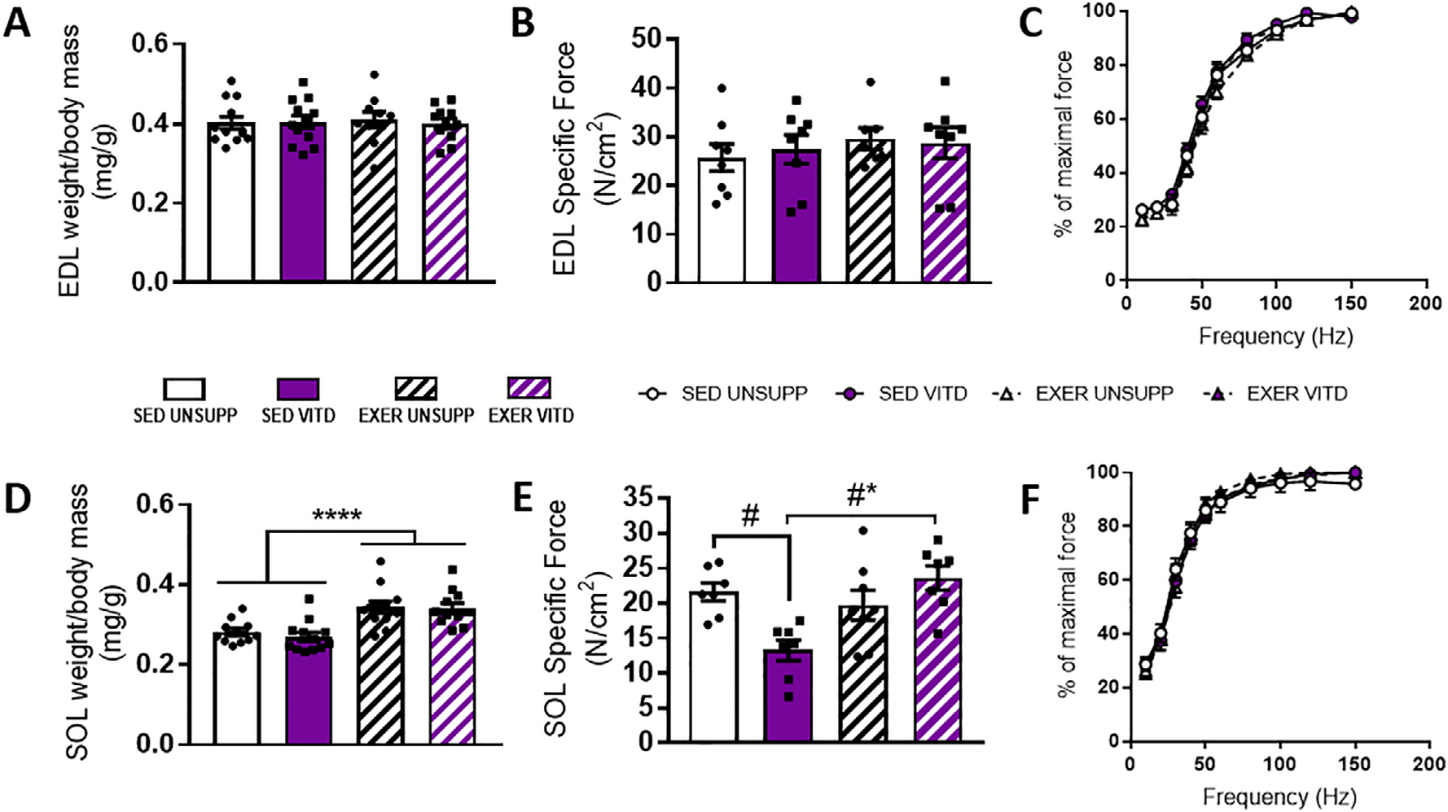
EDL and SOL muscle weight to body mass ratio, specific force production, and force‐frequency relationship of UNSUPP and VITD SED and EXER mice. EDL (*A*) and SOL (*D*) muscle mass to body mass ratios were calculated and are displayed. Force produced per CSA (specific force) for the EDL (*B*) and SOL (*E*). Force‐frequency relationship of the EDL (*C*) and SOL (*F*) was also determined and expressed as a percentage of absolute max force (Po). *p < .05 and ****p < .0001; EXER effect; #*p* < .05, VITD effect. *n* = 8 per group. EDL = extensor digitorum longus; EXER = exercise‐enriched; SED = sedentary; SOL = soleus; UNSUPP = vitamin D–unsupplemented; VITD = vitamin D–supplemented.

When muscle mass of the EDL and SOL was normalized to body mass, it was found that vitamin D has no influence on muscle mass (Fig. 5 *A*,*D*). Exercise enrichment, however, increased SOL mass (*p* < .0001, Fig. 5 *D*) but had no effect on the EDL. As summarized in Table 3, vitamin D increased P_t_ in the EDL (*p* < .05) but had no effect on any other contractile parameters measured in this muscle. However, there was a main effect of exercise enrichment to decrease P_t_/P_o_ (*p* < .01) and CSA (*p* < .05) in the EDL. In the SOL, exercise enrichment decreased P_t_/P_o_ (*p* < .05) overall and vitamin D decreased P_t_ and P_o_ between sedentary groups only (*p* < .05). However, when vitamin D and exercise enrichment were combined, P_t_ increased in the EXER VITD group compared to both EXER UNSUPP and SED VITD animals (*p* < .01).

**Table 3:** Comparison of Contractile Parameters Between All Groups

Parameter	SED UNSUPP	SED VITD	EXER UNSUPP	EXER VITD
EDL				
L_o_ (mm)	13.4 ± 1.0	13.8 ± 1.3	13.4 ± 0.9	13 ± 1.1
P_t_ (mN)	43.1 ± 15.3	45.1 ± 17.1#	29.7 ± 8.2**	35.2 ± 9.9**, #
P_o_ (mN)	179.06 ± 18.17	176.95 ± 18.41	172.54 ± 10.02	169.45 ± 15.71
P_t_/P_o_	0.28 ± 0.05	0.29 ± 0.05	0.20 ± 0.05**	0.25 ± 0.05**
TTP (ms)	17.1 ± 1.0	16.7 ± 1.0	15.2 ± 1.1	15.9 ± 1.1
½RT (ms)	12.4 ± 4.0	10.6 ± 3.5	7.5 ± 1.7	12.3 ± 6.3
CSA	0.0211 ± 0.0119	0.0211 ± 0.0031	0.0177 ± 0.0026**	0.0183 ± 0.0037**
Soleus				
L_o_ (mm)	12.3 ± 1.3	12.1 ± 1.2	13.4 ± 0.6	11.5 ± 1.0
P_t_ (mN)	24.9 ± 6.6	22.1 ± 12.6#	23.3 ± 7.9	27.2 ± 9.3**, #
P_o_ (mN)	112.84 ± 3.34	69.83 ± 8.83 #	105.89 ± 10.21	136.86 ± 8.84
P_t_/P_o_	0.26 ± 0.05	0.28 ± 0.03	0.22 ± 0.05*	0.24 ± 0.04*
TTP (ms)	23.3 ± 2.2	22.9 ± 1.62	23.5 ± 3.3	23.8 ± 2.64
½RT (ms)	14.2 ± 3.7	13.2 ± 7.2	15.2 ± 5.1	15.1 ± 7.74
CSA	0.0161 ± 0.0024	0.0153 ± 0.0017	0.0163 ± 0.0022	0.0174 ± 0.0027

All mean values displayed with ± SE. *n* = 7–10 per group. ½RT = one‐half relaxation time; CSA = cross‐sectional area; L_0_; = optimal length; Pt = twitch force; Po = peak tetanic force; Pt/P_O_ = twitch:tetanus ratio; TTP = time‐to‐peak tension.

* *p* < .05

** *p* < .001, exercise enrichment main effect.

# *p* < .05

VITD supplementation effect.

When P_o_ was corrected for force produced per CSA (specific force; sPo), there was no effect of either intervention in the EDL (Fig. 5 *B*). VITD had a more profound effect on sPo in the SOL, with a decrease in force production observed in the SED VITD when compared to the SED UNSUPP group (*p* < .01; Fig. 5 *E*). However, when vitamin D and exercise enrichment was combined, force production was increased (*p* < .05, Fig. 5 *E*).

### Force‐frequency relationship and muscle fatigue

**Figure 6:**
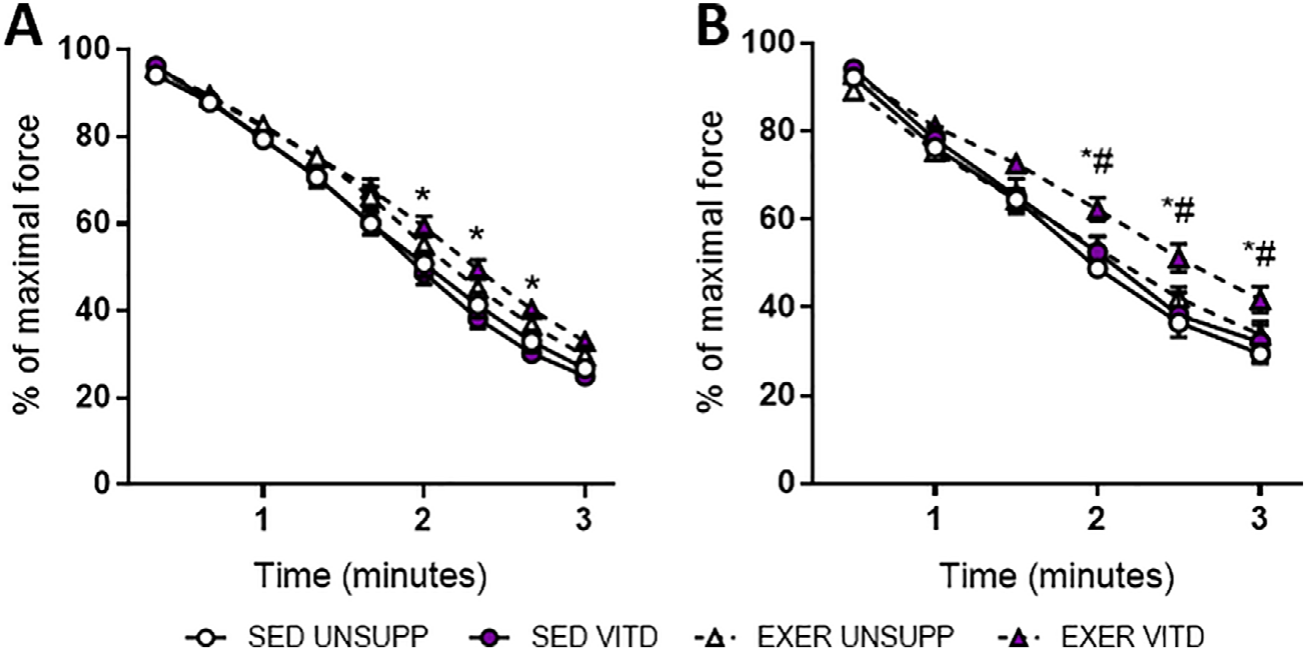
EDL and SOL ex vivo analysis of muscle fatigability of UNSUPP and VITD SED and EXER mice. Muscle fatigability for the EDL (*A*) and SOL (*B*). Symbols: open black circle, SED UNSUPP; full blue circle, SED VITD; open black triangle, EXER UNSUPP; full blue triangle, EXER VITD; *p < .05, between EXER VITD and SED VITD (exercise enrichment effect); #*p* < .05 between SED VITD and EXER VITD (vitamin D effect). *n* = 6–8 per group. EDL = extensor digitorum longus; EXER = exercise‐enriched; SED = sedentary; SOL = soleus; UNSUPP = vitamin D–unsupplemented; VITD = vitamin D–supplemented.

As shown in Fig. 5, it was found that neither vitamin D nor exercise enrichment affected the force‐frequency relationship curve for the EDL (Fig. 5 *C*) and SOL (Fig. 5 *F*) muscles. Surprisingly, fatigue resistance was not improved by exercise enrichment in UNSUPP animals in either the EDL or SOL (SED UNSUPP versus EXER UNSUPP; Fig. 6 *A*). However, in the SOL, fatigue resistance was improved in the EXER VITD group compared to all groups (*p* < .05; Fig. 6 *B*), highlighting a beneficial combination effect of vitamin D and exercise enrichment. Despite no beneficial effect of exercise enrichment on EDL fatigability in the UNSUPP mice (SED UNSUPP and EXER UNSUPP), when exercise enrichment was combined with vitamin D, improved force production during the later stages of the fatigue run was found (SED VITD versus EXER VITD; *p* < .05, Fig. 6 *A*).

### EDL and SOL histological analysis

**Figure 7:**
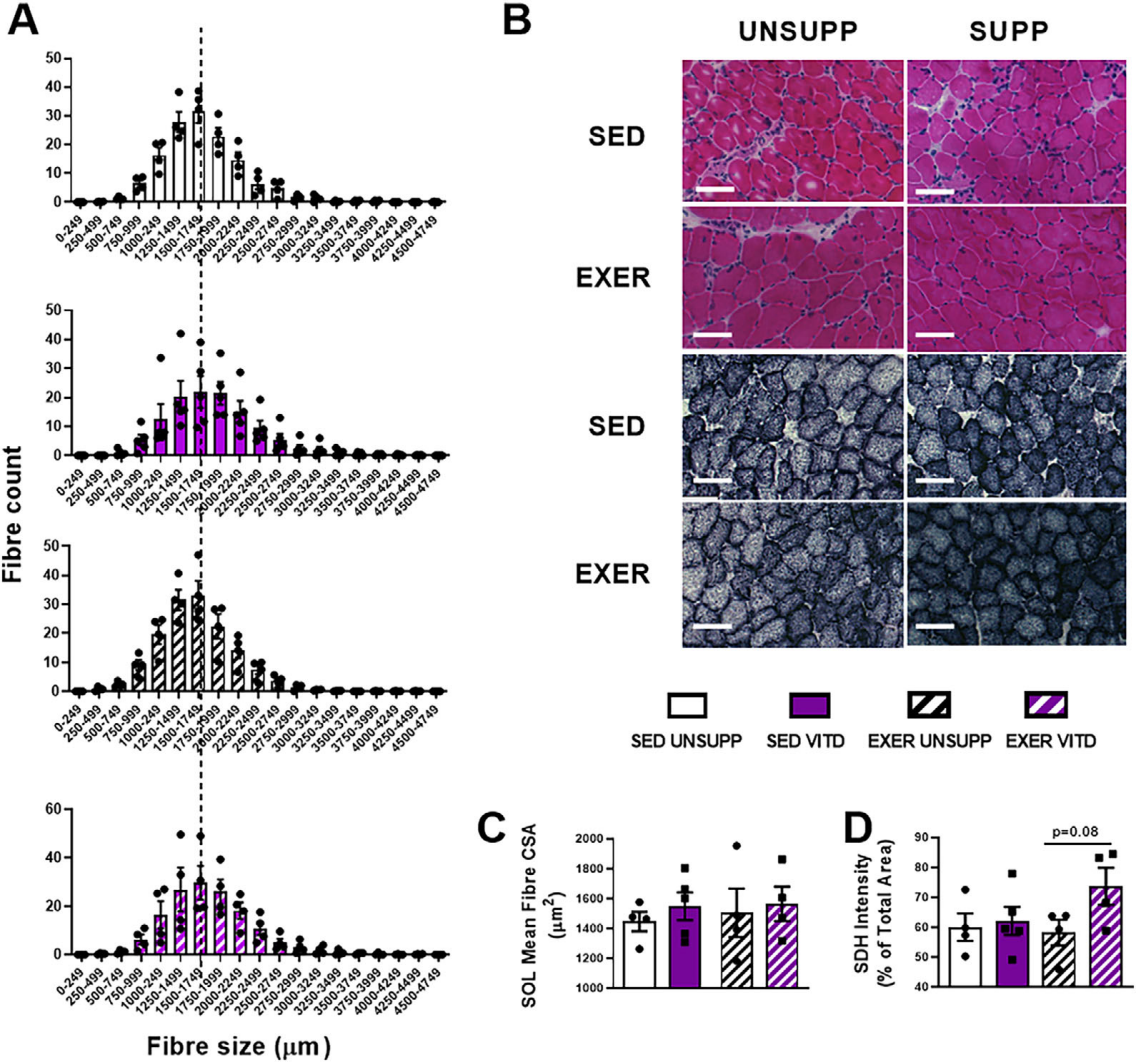
Histological and biochemical analysis of the SOL muscle of UNSUPP and VITD SED and EXER mice. H&E and SDH staining was performed on pre‐sectioned SOL tissue for general muscle architecture and mitochondrial content. (*B*) Representative images: H&E stain and SDH stain. Frequency of fiber distribution with average fiber area of control (SED UNSUPP) indicated with black broken line to show axis shift (*A*), mean fiber area (*C*), and SDH intensity (% of total area) (*D*). *n* = 4 per group. EXER = exercise‐enriched; H&E = hematoxylin and eosin; SDH = succinate dehydrogenase; SED = sedentary; SOL = soleus; UNSUPP = vitamin D–unsupplemented; VITD = vitamin D–supplemented.

**Figure 8:**
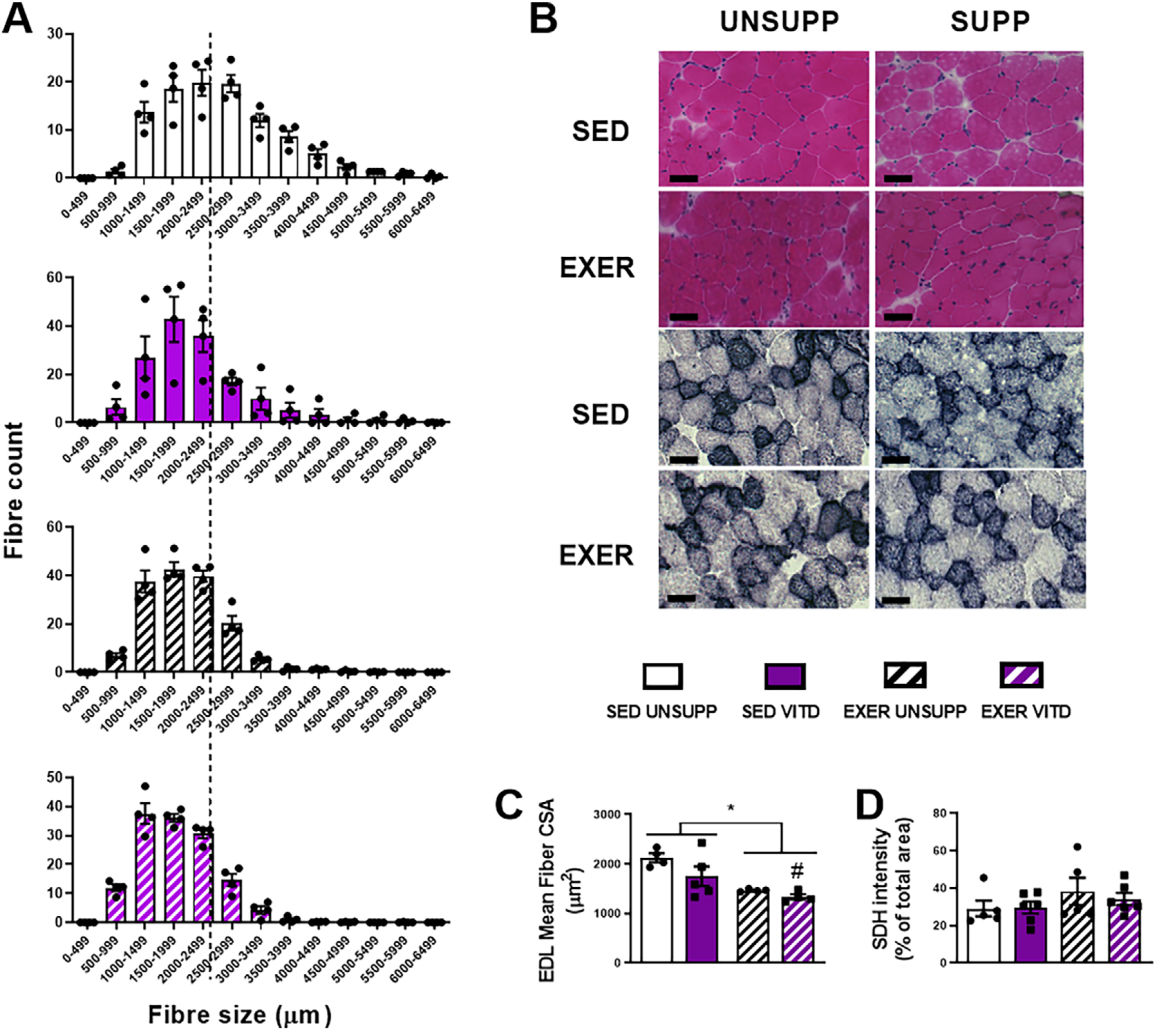
Histological and biochemical analysis of the EDL muscle of UNSUPP and VITD SED and EXER mice. H&E and SDH staining was performed on pre‐sectioned EDL tissue as a measure of general muscle architecture and mitochondrial content. (*B*) Representative images: H&E stain and SDH stain. Frequency of fiber distribution with average fiber area of control (SED UNSUPP) indicated with black broken line to show axis shift (*A*). Mean fiber area (*C*) and SDH intensity (% of total area) in the EDL muscle (*D*). Symbols: *p < .05, exercise enrichment effect; #*p* < .05, vitamin D effect. *n* = 4 per group. EDL = extensor digitorum longus; EXER = exercise‐enriched; H&E = hematoxylin and eosin; SDH = succinate dehydrogenase; SED = sedentary; UNSUPP = vitamin D–unsupplemented; VITD = vitamin D–supplemented.

To investigate the influence of vitamin D and/or exercise enrichment on morphological changes to the EDL and SOL muscles, H&E and SDH histological staining was conducted. In the SOL muscle, it was found that neither vitamin D nor exercise enrichment affected mean fiber CSA (see Fig. 7 *A*‐*C*). Surprisingly, there was no effect of exercise enrichment on SDH staining intensity in the SOL muscle, although this muscle is already highly oxidative in nature. A *t* test revealed a trend for vitamin D to increase SDH between the exercise‐enriched groups (*p* = .08, Fig. 7 *D*). In the EDL, there was a main effect of exercise enrichment to decrease mean fiber CSA when compared to sedentary animals (*p* < .05, Fig. 8 *A*‐*C*), with vitamin D contributing to a further decrease between EXER UNSUPP and EXER VITD groups only (*p* < .05, Fig. 8 *A*‐*C*). Overall, there was no effect of vitamin D or exercise enrichment on SDH staining intensity in the EDL (Fig. 8 *D*).

### CS activity and mitochondrial proteins

**Figure 9:**
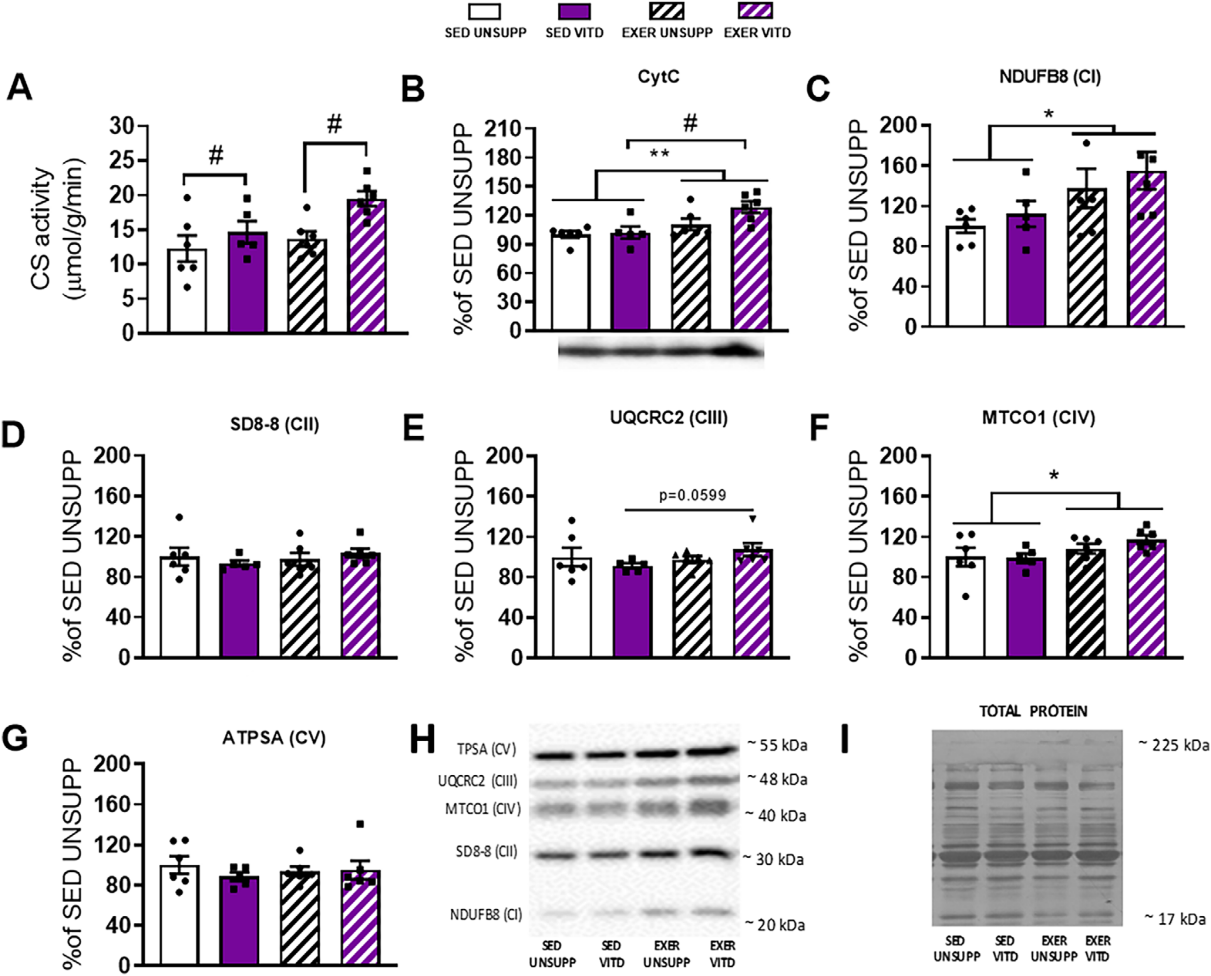
CS activity, CytC expression, and mitochondrial respiratory chain complex proteins of the TA muscle from UNSUPP and VITD SED and EXER mice. As a measure of mitochondrial capacity and quantity, CS activity (*A*), CytC protein expression (*B*) and mitochondrial respiratory chain complex proteins (*C‐G*) was investigated and displayed. Representative image of Western blots of proteins (*H*) with Coomassie blue stains (*I*) of the respective Western blots to demonstrate equal loading of the total protein as demonstrated by Timpani and colleagues^33^ in 2017. Symbols: **p < .01, exercise enrichment effect; #*p* < .05, vitamin D effect. *n* = 5–7 per group. CS = citrate synthase; CytC = cytochrome C; EXER = exercise‐enriched; SED = sedentary; TA = tibialis anterior; UNSUPP = vitamin D–unsupplemented; VITD = vitamin D–supplemented.

**Figure 10:**
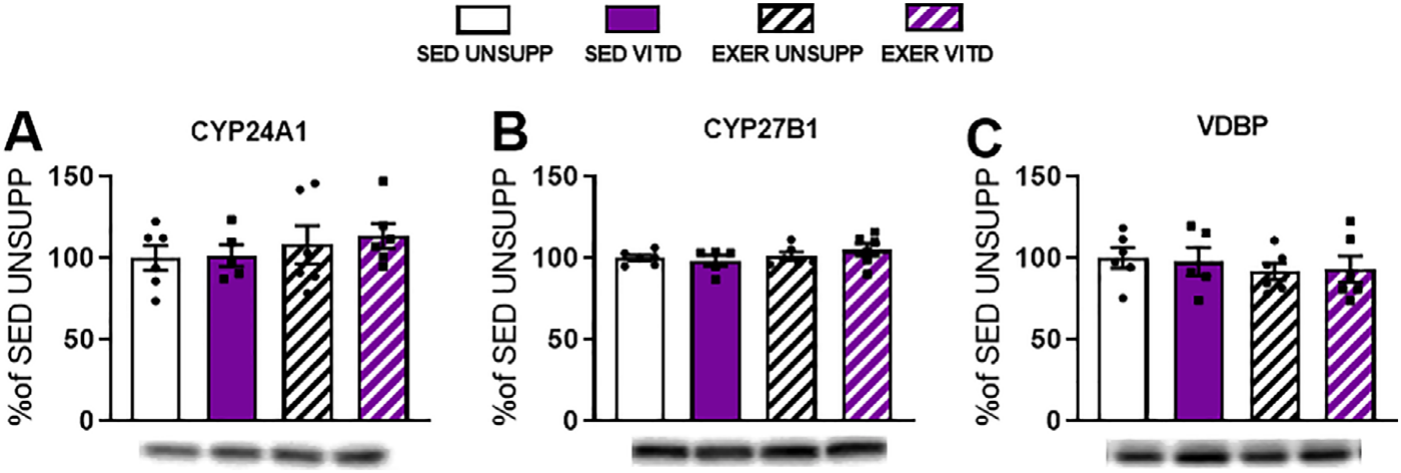
Vitamin D enzyme and carrier protein expression in the TA muscle of UNSUPP and VITD SED and EXER mice. 24α‐hydroxylase (CYP24A1, *A*), 1α‐hydroxylase (CYP27B1, *B*), and VDBP (*C*). All values were expressed as a percentage of control (SED UNSUPP). *n* = 5–6 per group. EXER = exercise‐enriched; SED = sedentary; TA = tibialis anterior; UNSUPP = unsupplemented; VDBP = vitamin D binding protein; VITD = vitamin D–supplemented.

Due to limited tissue remaining in the EDL, the TA muscle was used in an attempt to further elucidate and explain the changes in fatigability and morphological changes given it is found in the same compartment in the hindlimb and contains similar fiber‐type characteristics. With changes observed in muscle fatigability in both the fast twitch and slow twitch muscles in response to vitamin D and exercise enrichment combined, we decided to further investigate the role of the mitochondria in these changes. As shown in Fig. 9, vitamin D increased CS activity in both sedentary and exercise‐enriched groups when compared to UNSUPP animals (*p* < .05, Fig. 9 *A*). Exercise enrichment increased cytochrome C (CytC) expression in the UNSUPP animals and a combined effect of vitamin D and exercise enrichment was observed (*p* < .05, Fig. 9 *B*). Expression of five respiratory chain complex proteins of the mitochondria were also investigated and it was found that vitamin D had no effect (see Fig. 9 *C*–*G*). However, exercise enrichment increased expression of complex I and IV (*p* < .05, Fig. 9 *C* and *F*, respectively), with a trend to increase expression of complex III between SED VITD and EXER VITD groups only (*p* = .0599, Fig. 9 *E*). Furthermore, we decided to investigate whether changes are observed in the expression of two mitochondrial enzymes responsible for vitamin D metabolism namely, 24α‐hydroxylase and 1α‐hydroxylase. To our surprise, there was no effect of either intervention on the expression of either enzyme in the TA muscle (*p* > .05, Fig. 10 *A* and *B*, respectively). In addition to this, vitamin D protein levels also remained unchanged (*p* > .05, Fig. 10 *C*).

## Discussion

We hypothesized that increasing vitamin D levels above normal would improve aspects of skeletal muscle function. In particular, we examined whether high levels of vitamin D obtained from the diet would increase voluntary running distances, speed, and frequency of mice. We also investigated the effect of vitamin D on body composition, skeletal muscle function, and fatigue, both with and without voluntary exercise enrichment. This is the first study to combine high vitamin D supplementation with exercise to experimentally evaluate whether vitamin D alone, or together with exercise, can improve muscle function and/or exercise performance. Although vitamin D supplementation alone had no effect on voluntary running distance, increases in running speed were observed, which was associated with increased CS activity. Importantly, our data has demonstrated that in slow‐twitch SOL muscle, high vitamin D supplementation negatively impacts force output in sedentary animals. However, when supplementation was combined with exercise enrichment, not only was the negative impact of high vitamin D abolished, there were in fact noticeable improvements in force output and fatigability (ie, EXER VITD versus SED VITD mice). In addition to this, alterations to body fat mass and ex vivo muscle contractile function in the SOL were also found as a result of combining exercise with high vitamin D supplementation. Furthermore, this combination regimen increased SDH and CytC expression, further highlighting a potential link between high vitamin D, exercise, and the mitochondria.

It is well established that low or deficient levels of vitamin D are associated with muscle atrophy/low muscle mass and that repletion of vitamin D levels can effectively restore muscle mass, albeit via an unknown mechanism.^3^, ^10^ In this study, animals were supplemented with 20,000 IU/kg/diet vitamin D, which has been shown previously to elevate serum vitamin D levels by fivefold.^28^ Although serum vitamin D concentration was not measured in the current study (a notable limitation), the broad improvements in skeletal muscle function observed suggest comparable bioavailability of vitamin D metabolites. Our data shows that high levels of vitamin D may influence body composition with a strong trend for vitamin D supplementation to increase lean mass in sedentary mice (SED UNSUPP versus SED VITD, *p* = .051, Fig. 1 *C*). To take into account the variable amounts of vitamin D consumed by each animal, correlations were calculated between vitamin D intake and fat and lean mass index (Fig. 2). We expected to see an effect of exercise enrichment to alter body composition (increase lean mass and decrease fat mass) in the UNSUPP mice; however, we did not. Instead, we observed a strong relationship between vitamin D intake (SED VITD and EXER VITD groups) and body composition changes—the more vitamin D consumed in conjunction with exercise enrichment, the greater the lean mass and lower the fat mass. Although the reason for this is unclear, it is apparent that there is a complex interaction between high vitamin D and exercise, and it is possible that there is a sensitization to vitamin D caused by exercise, or vice versa. This requires further investigation.

Normalizing vitamin D levels in deficient individuals through supplementation has shown to increase muscle strength and performance.^4^, ^5^, ^13^, ^14^ Moreover, evidence of improved calcium handling, and increased aerobic capacity, muscle power and velocity, and improved postexercise recovery rates, suggests that vitamin D may be a useful ergogenic aid.^25^, ^26^, ^27^ Although these effects were demonstrated in deficient subjects, it is unclear whether these favorable adaptations would also occur in those with normal vitamin D levels. Given that no studies to date have combined high vitamin D supplementation with exercise, we introduced a voluntary running component during the 8‐week feeding period to assess whether vitamin D modified running performance. Contrary to our hypothesis, it was found that vitamin D supplementation did not affect voluntary running wheel distance or time spent on the wheel (Fig. 3 *B*,*C*); however, it did increase running speed (Fig. 3 *D*). Although vitamin D decreased voluntary activity in the first 4 days of the feeding period (*p* = .07, Fig. 3 *A*), this was quickly normalized such that there were no differences observed in average daily distances between groups. To further understand the effects of vitamin D on running performance, all mice were subject to metabolic cage screening at the end of the feeding and exercise enrichment protocol (Fig. 4). Interestingly, vitamin D decreased non‐wheel activity (pedestrian meters) regardless of exercise status (Fig. 4 *D*), suggesting that vitamin D may influence resting periods postexercise. This effect may be caused by factors that impact the central nervous system and behavior, which requires further investigation.

With regard to muscle contractile function, it was found that neither vitamin D nor exercise enrichment impacted twitch properties or half relaxation times (Table 3) suggesting that there was no effect of either intervention on intracellular calcium handling. Consistent with this, there was no effect of vitamin D or exercise enrichment on the generation of force at any particular frequency of activation (Fig. 5 *C*,*F*). Interestingly, absolute and specific force production in the SOL muscle was negatively impacted by vitamin D supplementation without exercise enrichment (SED UNSUPP versus SED VITD, Fig. 5 *E*). This finding might go toward explaining why the vitamin D–supplemented animals displayed an initial decline in pedestrian meters during Promethion metabolic screening (Fig. 4 *D*). Slow‐twitch muscles are more likely to be used during normal activity, as opposed to high‐velocity voluntary running, in which fast‐twitch muscles would dominate.^38^ Interestingly, there was a trend for vitamin D to decrease fat content within the SOL muscle (see Supplementary Fig. S1) in both supplemented groups. Although this may come as a surprise given the inverse relationship between intramuscular fat and strength, this suggests that perturbations in force production found in the sedentary supplemented animals are not due to fat infiltration, and are thereby due to another mechanism.

It has recently been speculated that high dosages of vitamin D may be harmful to muscle function, with a study reporting increased risk of falls in the elderly given a single dose of vitamin D that is equivalent to the average yearly intake (500,000 IU).^20^ This study speculated that decreased muscle strength may be a contributing factor, with the greatest decline seen in patients with fluctuating 25(OH)D serum levels from baseline.^39^ Interestingly, our recent study^22^ demonstrated that the more gradual introduction of high vitamin D over 4 weeks increased force production. However, the current study demonstrates that the same diet ingested over an 8‐week period decreased SOL muscle strength in SED VITD mice, perhaps suggesting that there is a threshold beyond which elevating vitamin D becomes deleterious. Although this clearly needs further investigation, it was surprising to note that this decrease in force production was effectively reversed with the addition of exercise enrichment (Fig. 5 *E*). Indeed, it is also interesting to note that there was a general decline in running performance in the initial 4 days of the high vitamin D diet, with EXER VITD mice covering significantly less distance compared to the EXER UNSUPP group at days 1 to 4 of wheel exposure (Fig. 3 *A*). This is perhaps suggestive of a decline in function with high vitamin D supplementation. However, no differences were present between groups at each time point thereafter. This reversal of the early decline in running performance and decreased force production suggests that exercise, in combination with vitamin D, may protect against functional declines in muscle function caused by high doses of vitamin D. Therefore, it is reasonable to speculate that if subjects treated with a single high annual dose of vitamin D were also prescribed regular exercise, then perhaps the observed increases in falls and fractures could be attenuated.

To further explore the different responses in the EDL and SOL, we conducted histological analysis. Although the SOL mass increased with vitamin D and exercise, we surprisingly did not observe a concomitant increase in mean fiber size (Fig. 7 *C*), nor any changes in calcium (Supplementary Fig. S1). We did, however, detect increased SDH intensity (Fig. 7 *D*), suggesting elevated oxidative capacity. In contrast to the SOL, and despite the presumably lower level of activation (as determined by no change in EDL mass), there were morphological changes observed in the EDL. Exercise enrichment decreased overall mean fiber size (Fig. 8 *C*), with the combination of vitamin D and exercise further decreasing mean fiber size (Fig. 8 *C*). Unlike the SOL, SDH intensity did not change in the EDL (Fig. 8 *D*). This suggests that there was no shift in the fiber type toward a more oxidative phenotype and does not explain the decreased fatigability observed in the EDL between SED VITD and EXER VITD groups (Fig. 6 *A*). Thus, the reason for decreased fatigability between SED VITD and EXER VITD groups remains to be determined but increased capillarization may play a role leading to enhanced oxygen delivery, enabling improvements in fatigue resistance.^40^ In addition to this, our data demonstrated an increase in CytC and complex III expression in the TA muscle (Fig. 9 *B*,*E*) between vitamin D supplemented groups only. Thus, we suspect that vitamin D may be contributing to alterations to the mitochondria found in the EDL, offsetting the effects of fatigue.

**Figure 11:**
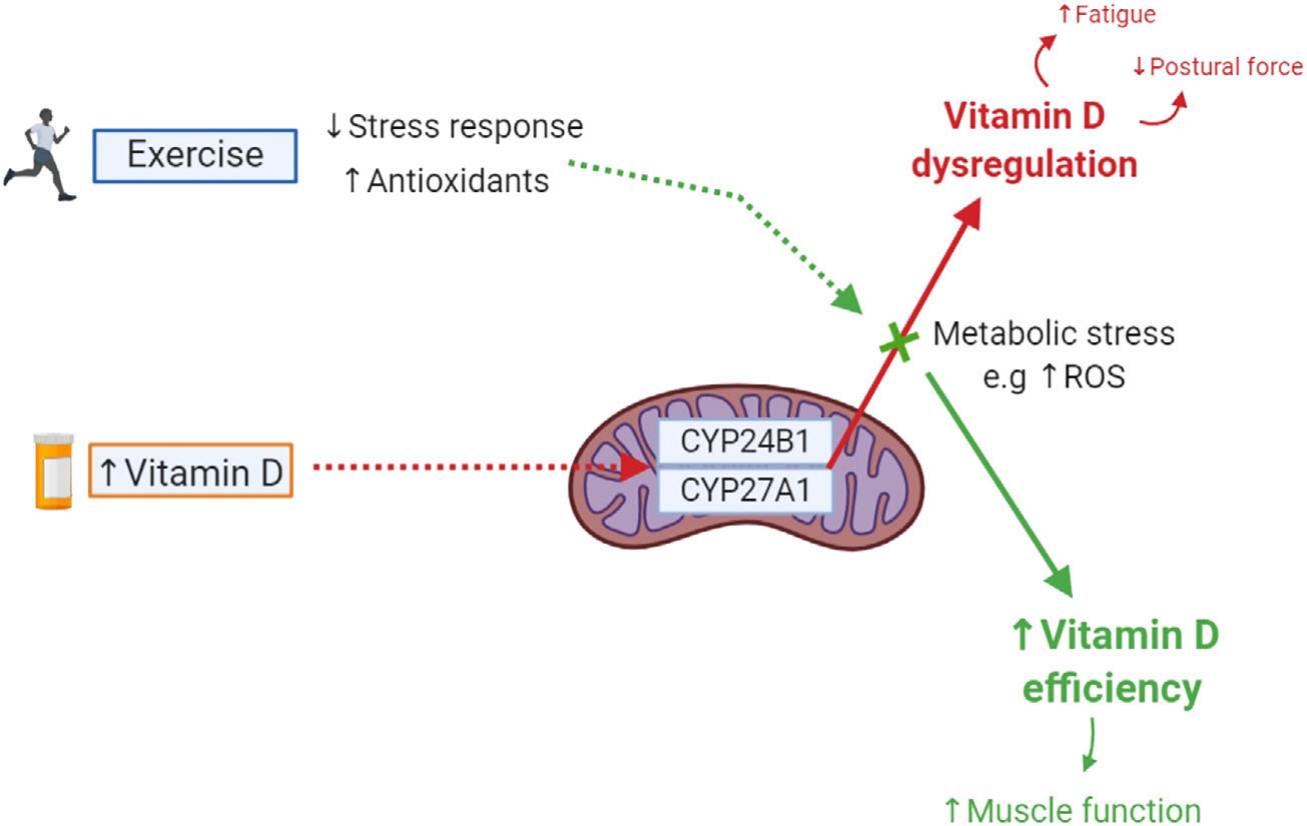
Our proposed mechanism of the reversed effects of vitamin D supplementation with exercise and the interplay it may have with mitochondrial enzyme activity and adaptations to metabolic stressors induced by exercise training. Here, we suspect that administration of vitamin D in large amounts results in dysregulation of vitamin D levels. We speculate that this alteration is caused by perturbations in the activity of key metabolic enzymes, 1,25‐alpha‐hydroxylase (CYP27A1) and 24‐alpha‐hydroxylase (CYP24B1). Due to beneficial adaptations in the mitochondria seen with exercise training, we suspect that exercise may be modulating and/or reducing the metabolic stress response caused by high dose vitamin D supplementation, resulting in vitamin D efficiency and improvements in muscle function. ROS = reactive oxygen species.

Although we only saw the effects of exercise in the SOL of supplemented mice, we suspect that there is a strong interplay between vitamin D, exercise, and the mitochondria. In particular, the key role of metabolic enzymes, such as 1,25‐α‐hydroxylase and 24‐α‐hydroxylase, makes it an attractive target of interest. We decided to investigate the expression of CYP27A1 and CYP24B1 enzymes and the vitamin D binding protein (VDBP) in the readily available and prepared TA muscle. Although no effect of either intervention was demonstrated (Fig. 10), it is reasonable to speculate that adaptations to these regulatory enzymes or other processes may have occurred at a different time point during the intervention and future studies should take this into consideration. Indeed, we speculated that 24‐α‐hydroxylase, which is responsible for catabolism of vitamin D and inactivation of conversion enzyme 1,25‐α‐hydroxylase, overactivates in response to high levels of vitamin D and metabolic stress due to its homeostatic or “protective” nature.^41^, ^42^ It may be possible that inhibition of 1,25‐α‐hydroxylase, in an attempt to lower vitamin D, results in vitamin D dysregulation and subsequent alterations to the force‐generating ability of the muscle. In fact, it is possible that this mechanism could also help explain the increased risk of falls in human studies administering yearly doses. However, exercise may have been able to reverse or prevent mechanisms contributing to decreased muscle function and improve fatigue resistance likely due to decreasing metabolic stress responses through mitochondrial adaptations (such as increasing antioxidants). Simply put, we suspect exercise alleviates the increased activity of 24‐α‐hydroxylase and alters mitochondrial function thus contributing to the prevention of vitamin D dysregulation and subsequent decreased muscle function. A summary of this proposed mechanism can be found in Fig. 11.

In conclusion, this study is the first to show a beneficial effect of exercise enrichment and vitamin D on skeletal muscle function. Although the mechanisms remain unclear, it appears that these effects may be fiber type–specific, with the most profound effect displayed in the slow‐twitch SOL postural muscle. These findings may help explain the increased rates of falls and fracture in elderly patients when given high bolus amounts of vitamin D. Furthermore, these findings suggest that exercise is protective against the negative side effect of high vitamin D and should be incorporated into regimens where prescription of high vitamin D supplementation is prescribed.

## Disclosures

All authors declare no conflicts of interest.

## Supporting information


**Supplementary Fig. S1.** Histological analysis of calcification and fat content. To detect whether high vitamin D causes calcification in the muscle, the soleus was cut and stained with Alizarin Red. There was no effect of either vitamin D supplementation with or without exercise on calcification in the SOL (A). Intermuscular fat content was quantified via Oil‐Red‐O staining. Interestingly, there was a strong trend for vitamin D to decrease muscle fat content in sedentary and exercised animals (*p* = .064, B) with no effect of exercise in unsupplemented groups. Scale bar on all representative images = 100 μm. *n = 4* per group.

Supplementary Fig. S2.
