# Supplementary material for: Exercise May Ameliorate the Detrimental Side Effects of High Vitamin D Supplementation on Muscle Function in Mice
Source: J Bone Miner Res. 2020 Mar 11;35(6):1092–106. doi: 10.1002/jbmr.3985 (PMC9327727; doi:10.1002/jbmr.3985)
Supplement: Supplementary file 1 — Supplementary Fig. S1. Histological analysis of calcification and fat content. To detect whether high vitamin D causes calcification in the muscle, the soleus was cut and stained with Alizarin Red. There was no effect of either vitamin D supplementation with or without exercise on calcification in the SOL (A). Intermuscular fat content was quantified via Oil‐Red‐O staining. Interestingly, there was a strong trend for vitamin D to decrease muscle fat content in sedentary and exercised animals (p = .064, B) with no effect of exercise in unsupplemented groups. Scale bar on all representative images = 100 μm. n = 4 per group. [file JBMR-35-1092-s001.docx]

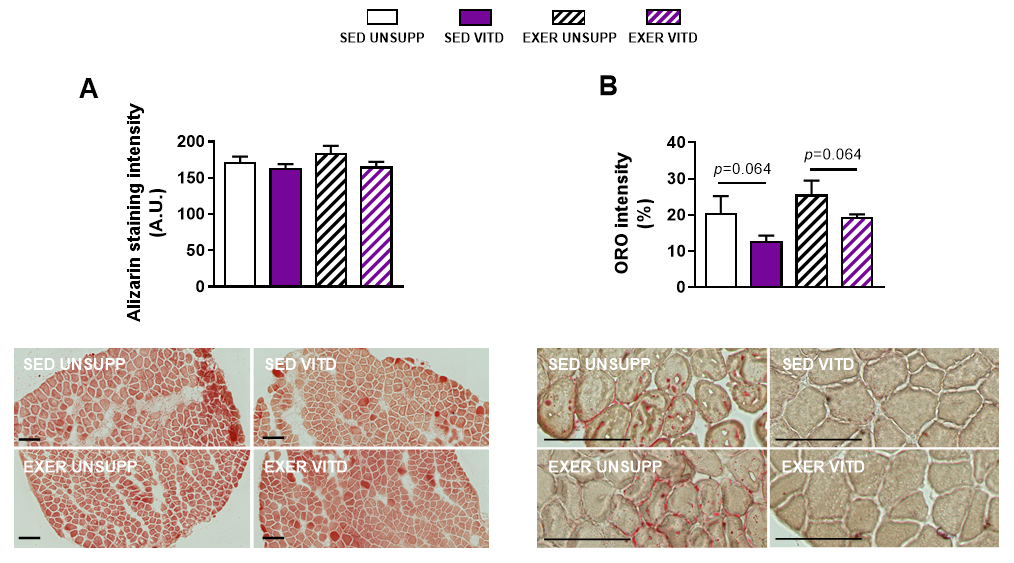


**Supplementary figure 1. Histological analysis of calcification and fat content.** To detect whether high vitamin D causes calcification in the muscle, the soleus was cut and stained with Alizarin Red. There was no effect of either vitamin D supplementation with or without exercise on calcification in the SOL (A). Intermuscular fat content was quantified via Oil-Red-O staining. Interestingly, there was a strong trend for vitamin D to decrease muscle fat content in sedentary and exercised animals (p=0.064, B) with no effect of exercise in unsupplemented groups. Scale bar on all representative images = 100μm. *n=4* per group
